# Causes, factors, and complications associated with hospital admissions among patients with Parkinson's disease

**DOI:** 10.3389/fneur.2023.1136858

**Published:** 2023-03-07

**Authors:** Navena Sharma Shaibdat, Norfazilah Ahmad, Shahrul Azmin, Norlinah Mohamed Ibrahim

**Affiliations:** ^1^Department of Medicine, Hospital Canselor Tuanku Muhriz, Faculty of Medicine, Universiti Kebangsaan Malaysia, Kuala Lumpur, Malaysia; ^2^Department of Community Health, Faculty of Medicine, Universiti Kebangsaan Malaysia, Kuala Lumpur, Malaysia

**Keywords:** Parkinson's disease, causes, factors, emergency, elective, hospital admission, deaths, complication

## Abstract

**Introduction:**

Patients with Parkinson's disease (PD) are at a higher risk of hospital admissions compared to the general population. We studied the causes and factors associated with admissions among patients with PD over 6 years.

**Methods:**

We included all PD admissions between 1 January 2016 and 31 December 2021. Other causes of parkinsonism were excluded. Causes of admissions were divided into PD-related (direct or indirect) or non-PD-related. The type of admission was categorized into emergency or elective.

**Results:**

We identified 605 hospital admissions (259 patients with PD); 345 (57.0%) were PD-related and 260 (43%) were non-PD-related. Emergency PD admissions contributed to 50.4% of all admissions, most commonly from respiratory infection (23%). PD admissions in comparison to non-PD admissions were associated with worse disease severity (HY ≥ 3; *p* < 0.001), longer disease duration [8.71 (SD 6.23) vs. 6.60 (SD 5.39) years; *p* < 0.001], and longer hospital stay [7.70 (SD 5.89) vs. 6.42 (SD 7.63) days; *p* = 0.020]. Non-PD admissions were associated with more comorbidities (97.3%; *p* = 0.013). There were 124 (20.5%) complications and 31 deaths (5.1%). A total of 29 deaths were due to respiratory infection and 3 deaths were due to COVID-19 pneumonia. Emergency admission (PD- and non-PD-related; *p* = 0.001) and respiratory-related causes (*p* < 0.001) were predictors of unfavorable hospital admission outcomes (death and complications).

**Conclusion:**

Respiratory infection was the leading cause of hospital admission and a significant independent predictor of unfavorable hospital admission outcomes (death and complications). PD-related admissions were associated with disease severity and led to more complications and longer hospital stays. Non-PD-related admissions were associated with comorbidities.

## Introduction

Parkinson's disease (PD) is a chronic and progressive neurodegenerative disease characterized by motor and non-motor symptoms, with marked heterogeneity in the clinical manifestation, treatment response, and disease progression, leading to variable courses of disease between individuals (1). While the use of levodopa has contributed to significant improvement in quality of life with increased life expectancy, the disease runs a more prolonged course resulting in significant morbidity in the long term (1). The Global Burden of Disease, Injuries and Risk Factors Study (GBD) 2015 reported that the number of people affected by PD had increased by 118% from 1990 to 2015 with 6.2 million people affected globally (2). By 2030, it is estimated that the number of patients will increase further to 9.3 million (3). The number of PD cases in Malaysia has also increased by 26.4% from 15,697 cases in 1990 to 23,908 in 2015 (4) and is expected to rise further with the rise in the aging population (3).

The World Health Organization has highlighted wide gaps in the care of patients with PD, one of which is the lack of effective therapies to prevent or improve both long-term motor and non-motor complications (1). With the rising prevalence of PD and increased life expectancy, it is anticipated that more patients with PD will require hospitalization for the management of their motor and non-motor symptoms. In addition, patients with PD are also susceptible to hospitalization due to comorbid diseases, given that most of them are elderly patients and frequently have other health problems (5). Hospitalization of patients with PD significantly impacts their wellbeing adding to the morbidity associated with the disease and may alter the disease course adversely (6). Admissions due to PD also led to higher health utilization costs compared to age-matched controls, thus contributing to a significant economic burden to patients and the healthcare system (7).

Various factors contribute to admissions among patients with PD. Some factors are directly related to the disease (PD-related), such as worsening of motor and non-motor symptoms, while others may be due to the presence of comorbid diseases (non-PD-related). It is also unclear how the COVID-19 pandemic impacts hospitalizations among patients with PD. Thus, understanding the frequent causes of hospitalization in PD is important in planning healthcare resources and in determining avoidable causes. Apart from a few studies conducted in India [10, 15], there is a lack of data on the causes of hospitalization in Asian countries, especially within the South East Asian region.

## Objective

This study was, therefore, conducted to determine the causes, factors, trends, and complications of admissions, including admissions during the COVID-19 pandemic years, among patients with PD to gain an understanding of the preventive strategies that could be instituted to reduce future hospitalizations.

## Methods

### Study setting and population

This was a retrospective review (cross-sectional design) of all PD admissions to Hospital Canselor Tuanku Muhriz (HCTM) from 1 January 2016 and 31 December 2021. HCTM is a tertiary hospital and a teaching center with 1,054 beds. The neurological services include an established movement disorder service offering comprehensive medical and surgical therapy for PD. Study approval was obtained from the UKM Medical Research Ethics Committee (research code: JEP-2021-263). We included all admissions of patients with idiopathic PD, with a minimum admission duration of 24 h. Patients who were diagnosed with secondary parkinsonism, Parkinson's Plus syndrome, an unclear diagnosis of idiopathic PD, and records with missing data such as staging of PD or duration of PD, were excluded from this study. The sample size was calculated using a sample size calculator for estimations (8, 9), at a 5% significance level, study power of 80%, and with reference to a previous study (10). The present study required a minimum sample size of 166.

### Data collection

Information on hospital admissions was obtained from the Case-Mix Unit of HCTM. Case-Mix is a classification of services implemented in diagnosis, treatment, and care in clinical terms (11, 12). Medical case notes of all hospital admissions with the International Classification of Diseases (ICD-10) code G20 (PD) (13) from 1 January 2016 to 31 December 2021 were identified. Data from the patient's medical case notes were retrospectively reviewed and entered into a datasheet which consisted of three sections: Section A: sociodemographic characteristics (age, gender, and race), Section B: clinical parameters (co-morbidities, staging of PD, duration of PD, age of PD onset, and previous admissions during the 6-year study period), and Section C: admission-related parameters (year of admission, causes of admission, length of hospital stay, complications encountered during hospital admission, and type of admission). For patients with more than one admission within the study duration, the demographic details were recorded for the most recent admission and prior admissions were referred to as previous admissions (10); each admission was recorded as a separate event. If patients had more than one admission during the study period, the previous admissions were categorized as repeated admissions.

#### Comorbidities

Comorbidities were grouped into five categories, namely, medical only; medical and orthopedics; medical and psychiatric; medical and others; and more than two comorbidity categories, based on the comorbidity trend among our study population. The following describes the diseases for each category.

- Medical only (hypertension (HPT), dyslipidemia, diabetes mellitus (DM), [with or without target organ damage such as ischemic heart disease, chronic kidney disease, end-stage renal disease, and stroke), bronchial asthma (BA), and chronic obstructive pulmonary disease (COPD)]
- Combined medical with orthopedic diseases (cauda equina syndrome, degenerative disc disease with or without spine surgery, spine fracture, long bone fracture, and after total knee replacement)
- Combined medical and psychiatric diseases (dementia, mood disorder, and psychosis)
- Combined medical and other comorbidities such as benign prostatic hyperplasia (BPH) and kidney/bladder stones
- Any combination of more than two comorbidity categories listed earlier.

#### Disease parameters

The Hoehn and Yahr (HY) staging was used for PD severity (14) and patients were divided into two groups based on HY stage, namely, HY 1-2 and HY ≥3. Duration of PD was classified into < 5 years, 5–10 years, and >10 years.

#### Admission-related

The type of admission was categorized into emergency (any admissions which went through the emergency department first, prior to ward admission) or elective (any admission that was planned from the outpatient department).

Causes of admission were divided into PD-related or non-PD-related causes.

Parkinson's disease-related causes were further divided into direct PD-related and indirect PD-related causes (10, 15).

- Direct PD-related causes include motor, non-motor, neuropsychiatric symptoms, and medication-related (adverse effects) and planned admissions for medication adjustment, levodopa challenge procedure, brain imaging, and DBS (deep brain stimulation) procedure.
- Indirect PD-related causes include infections such as all respiratory infections (community-acquired pneumonia, aspiration pneumonia, orthostatic pneumonia, and COVID-19 pneumonia), hospital-acquired infection, urinary tract infection, gastrointestinal infection, and infected pressure sore), trauma due to fall and any complications arising from the fall (fracture or intracerebral bleed), delirium, electrolyte imbalances, and all others that included infected PEG tube.

Non-PD-related causes include all other admissions related to the presence of comorbidities, which were unrelated to PD.

Following data collection, to facilitate analysis, we further categorized the causes of non-PD emergency admissions into five categories based on the top common causes from our study: cardiac causes, end-stage renal disease, stroke, anemia, and infection/metabolic causes, while non-PD elective admissions were categorized as planned orthopedic procedures or non-orthopedic procedures.

### Statistical analysis

Data were analyzed using the Statistical Package for the Social Sciences (SPSS) software version 26.0, IBM SPSS Statistics (RRID:SCR_019096). For descriptive analysis, categorical data were presented as frequency (n) and percentage (%). Mean and standard deviation (SD) was used for normally distributed continuous data. Pearson chi-square test was used to determine the association between causes of admission (PD-related and non-PD-related) and categorical variables, namely, age group, gender, race, comorbidities, staging of PD, duration of PD, and previous admissions and complications. *T*-test was used to determine the association between the mean age of PD onset, mean duration of PD, mean length of stay, and causes of admission (PD-related and non-PD-related). Multivariable logistic regression analysis was performed to assess factors that predict hospital admission outcomes. A significant level is set at *p* < 0.05.

## Results

**Figure 1:**
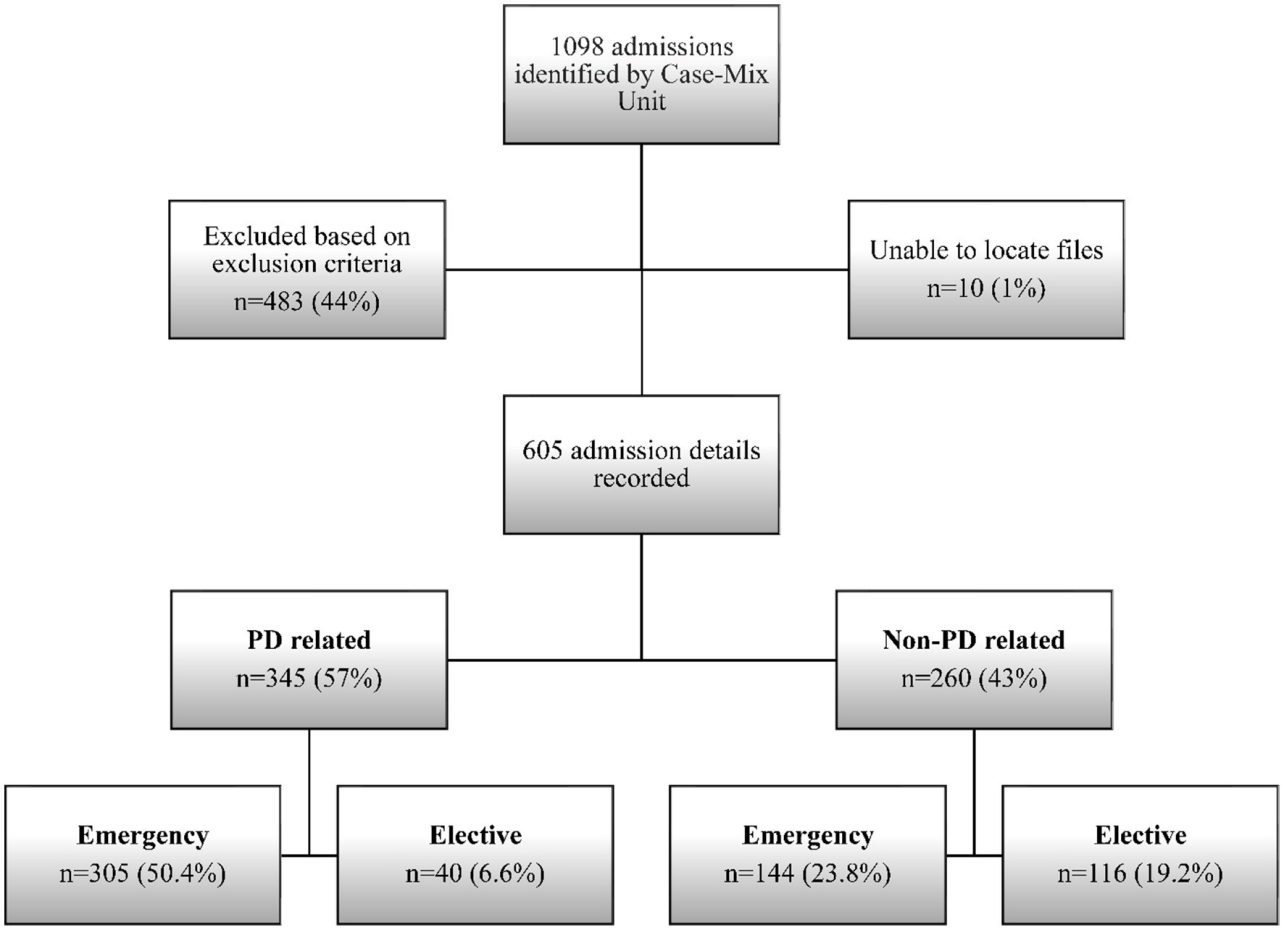
Study flow showing the different types of admissions.

A total of 1,098 admissions with a diagnosis of parkinsonism from a cumulative adult admission of 193,646 were identified by the Case-Mix Unit from 1 January 2016 to 31 December 2021. Of these, 21 had missing data and had to be excluded. Finally, only 605 admissions involving 259 patients with PD fulfilled the inclusion and exclusion criteria and were enrolled in the study (Figure 1). The estimated rate of PD admission based on the cumulative admissions over the 6-year period was 0.31%.

The mean age of patients with PD from this study was 72.4 (SD 8.84) years, and 98.5% were ≥ 50 years of age. There were 145 (56.0%) men and 114 (44.0%) women. Patients were mostly of Chinese ethnicity (60.6%), followed by Malay and Indian. The majority of patients (*n* = 171, 66.0%) were in the HY stage ≥ 3. Most patients (39.8%, *n* = 103) had a PD duration between 5 and 10 years, while 32.0% (*n* = 83) had a disease duration of < 5 years, and 28.2% (*n* = 73) had a disease duration of >10 years. Almost all patients (95%) had comorbidities; medical disorders being the most common (95%), followed by psychiatric and orthopedic disorders. Only 13 (5%) did not have any comorbid diseases (Table 1).

**Table 1:** Sociodemographic characteristics and clinical parameters of patients with PD (*n* = 259).

		***n* (%)**
Age (mean, years)		72.41 (SD 8.84)
	< 50	4 (1.5)
	≥50	255 (98.5)
Gender		
	Male	145 (56.0)
	Female	114 (44.0)
Race		
	Malay	87 (33.6)
	Chinese	157 (60.6)
	Indian	15 (5.8)
	Others	0
Comorbidities		
	Medical^a^	158 (61.0)
	Medical^a^ and orthopaedic^b^	34 (13.1)
	Medical^a^ and psychiatry^c^	36 (13.9)
	Medical^a^ and others^d^	9 (3.5)
	Combination of ≥ 2 co-morbids	9 (3.5)
	None	13 (5.0)
Hoehn Yahr Stage	1 & 2	88 (34.0)
	≥3	171 (66.0)
Duration of PD (years)	< 5	83 (32.0)
	5–10	103 (39.8)
	>10	73 (28.2)
Age of PD onset (mean, years)		64.37 (SD 10.70)

^a^Medical: DM ± HPT ± dyslipidemia ± TOD (IHD, CKD, ESRF, stroke) ± BA/COPD.

^b^Orthopedic: cauda equina syndrome, degenerative disc disease ± spine surgery, spine fracture, long bone fracture, post TKR.

^c^Psychiatry: dementia, mood disorder, psychosis.

^d^Others: BPH, kidney/bladder stones, ICB post fall.

SD, standard deviation; DM, diabetes mellitus; HPT, hypertension; TOD, target organ damage; IHD, ischemic heart disease; CKD, chronic kidney disease; ESRF, end stage renal failure; BA, bronchial asthma; COPD, chronic obstructive pulmonary disease; TKR, total knee replacement; BPH, benign prostatic hyperplasia; ICB, intracerebral bleed.

### Causes and types of admissions

Of the 605 admissions recorded over the 6-year period, 345 (57.0%) admissions were due to PD-related causes, while 260 (43.0%) admissions were non-PD-related. The frequency of the different types of admission is shown in Figure 1. For PD-related admissions, 305 (88.4%) were emergency admissions and 40 (11.6%) were elective admissions (Table 2). For non-PD-related admissions, 144 (55.4%) were emergency admissions and 116 (44.6%) were elective admissions.

**Table 2:** Causes and type of PD admissions.

**PD-related causes** ***n*** = **345 (57.0%)**				**Non-PD-related causes** ***n*** = **260 (43.0%)**			
**Emergency** ***n*** = **305 (88.4)**		**Elective** ***n*** = **40 (11.6)**		**Emergency** ***n*** = **144 (55.4)**		**Elective** ***n*** = **116 (44.6)**	
**Direct PD-related** ***n*** **(%)**				Cardiac	32 (22.2)	Ortho procedures^b^	45 (38.8)
Motor symptoms	24 (7.9)	Procedures^a^	33 (82.5)	End stage Renal disease	2 (1.39)	Non-ortho procedures^c^	71 (61.2)
Non motor symptoms	10 (3.3)	Medication adjustment	7 (17.5)	Stroke	11 (7.6)		
Neuro-psychiatric symptoms	2 (0.7)			Anemia (bleed, chronic illness)	29 (20.1)		
Medication adverse effects	16 (5.2)			Infection/Metabolic (catheter, gangrene, abscess)	70 (48.6)		
**Indirect PD-related**							
Sepsis (total)	199 (65.2)						
*Respiratory*	139 (45.6)						
*Genito-urinary*	39 (12.8)						
*Gastro-intestinal*	4 (1.3)						
*Pressure sores*	17 (5.6)						
Trauma (fall, fracture)	43 (14.1)						
Delirium	2 (0.7)						
Electrolyte imbalance	4 (1.3)						
Others^d^	5 (1.6)						

^a^Procedures: levodopa challenge, brain imaging.

^b^Orthopedic procedures such as nerve block, imaging, and surgery.

^c^Non-orthopedic procedures such as catheter insertion for dialysis, fistula creation, cataract surgery, and angiogram.

^d^Others; PEG tube infection.

Infection/sepsis (*n* =199, 65.2%) was the most common cause of PD-related emergency admissions, mostly due to respiratory infections (*n* = 139, 45.6%) such as community-acquired pneumonia (n = 60), aspiration pneumonia (*n* = 31), hospital-acquired pneumonia (*n* = 22), orthostatic pneumonia (*n* = 19), and COVID-19 pneumonia (*n* = 7). This was followed by genitourinary infection (*n* = 39, 12.8%). Trauma (14.1%) including its resulting complications was the second most common cause of PD-related admissions. PD-related planned procedures (levodopa challenge, brain imaging, and DBS insertion) were the third most common cause. The causes for non-PD-related emergency admissions in the order of highest frequency were infection/metabolic followed by anemia (gastrointestinal bleeding or chronic disease), cardiac disease, stroke, and renal disease. Elective non-PD-related admissions were either due to orthopedic procedures (38.8%) or other planned medical or surgical procedures (61.2%). Table 2 shows the causes of all admissions.

### Previous admissions

**Figure 2:**
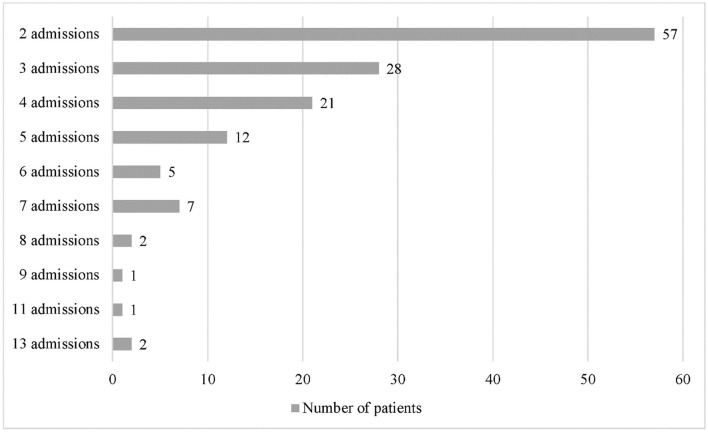
Frequency of repeated admissions.

Previous admissions are repeated admissions encountered by a single patient in the 6-year study period. Of the 605 admissions recorded, 482 (80.0%) were repeated admissions, which occurred in 136 out of 259 patients. The number of repeated admissions ranged from 2 to 13 admissions per patient (Figure 2). The causes for repeated admissions are shown in Supplementary Table 1.

**Figure 3:**
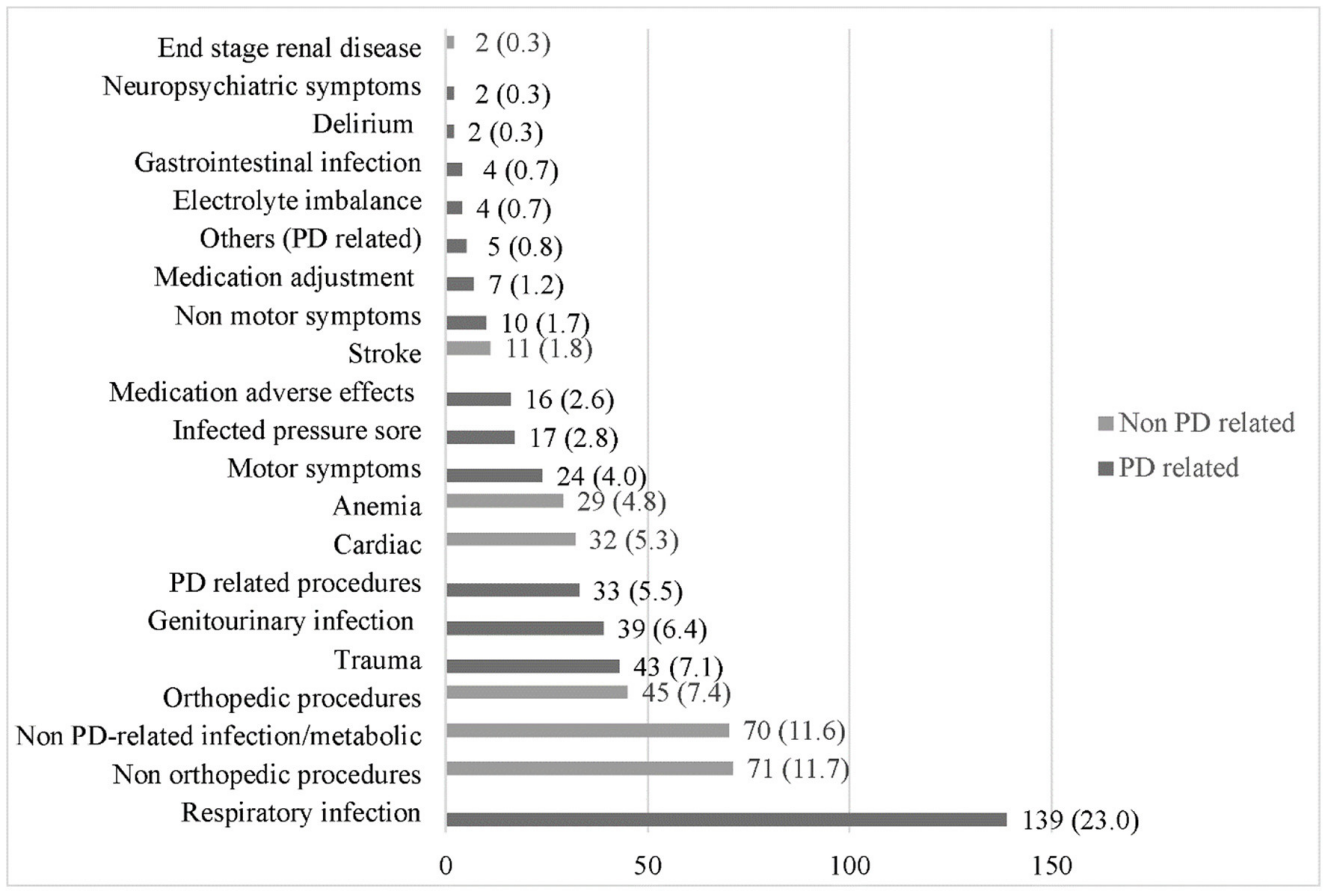
Frequency of causes of admission for PD and non-PD combined. Non-orthopedic procedures: catheter insertion, fistula creation, angiogram, cataract surgery. Non-PD-related infection/metabolic: catheter related blood stream infection, gangrene, abscess, uncontrolled diabetes. Orthopedic procedures: nerve block, surgery, imaging. PD-related procedures: imaging, levodopa challenge test, DBS insertion. Others (PD-related): infected PEG tube.

The causes of admissions irrespective of whether PD-related or non-PD-related are shown in Figure 3. The top five causes of admissions were respiratory infection (23%), followed by non-orthopedic planned procedures (11.7%), infection or metabolic causes unrelated to PD (11.6%), orthopedic planned procedures (7.4%), and trauma (7.1%).

### COVID-19 admissions

Between 2020 and 2021, there were only seven admissions, all in the year 2021 among PD patients with COVID-19 pneumonia who fulfilled the inclusion and exclusion criteria. There were three admissions in the year 2020 that were excluded from this study and not analyzed due to missing data. The mean age of the patients with COVID-19 pneumonia was 73.71 (SD 6.63) years and the mean PD duration was 6 (SD 3.79) years. All seven patients had comorbidities. Four patients were in HY stage ≥3. Of the seven admissions, three (42.9%) resulted in death, while four (57.1%) had complications such as hospital-acquired infection, delirium, and deterioration in clinical staging.

### Trend of admission

**Figure 4:**
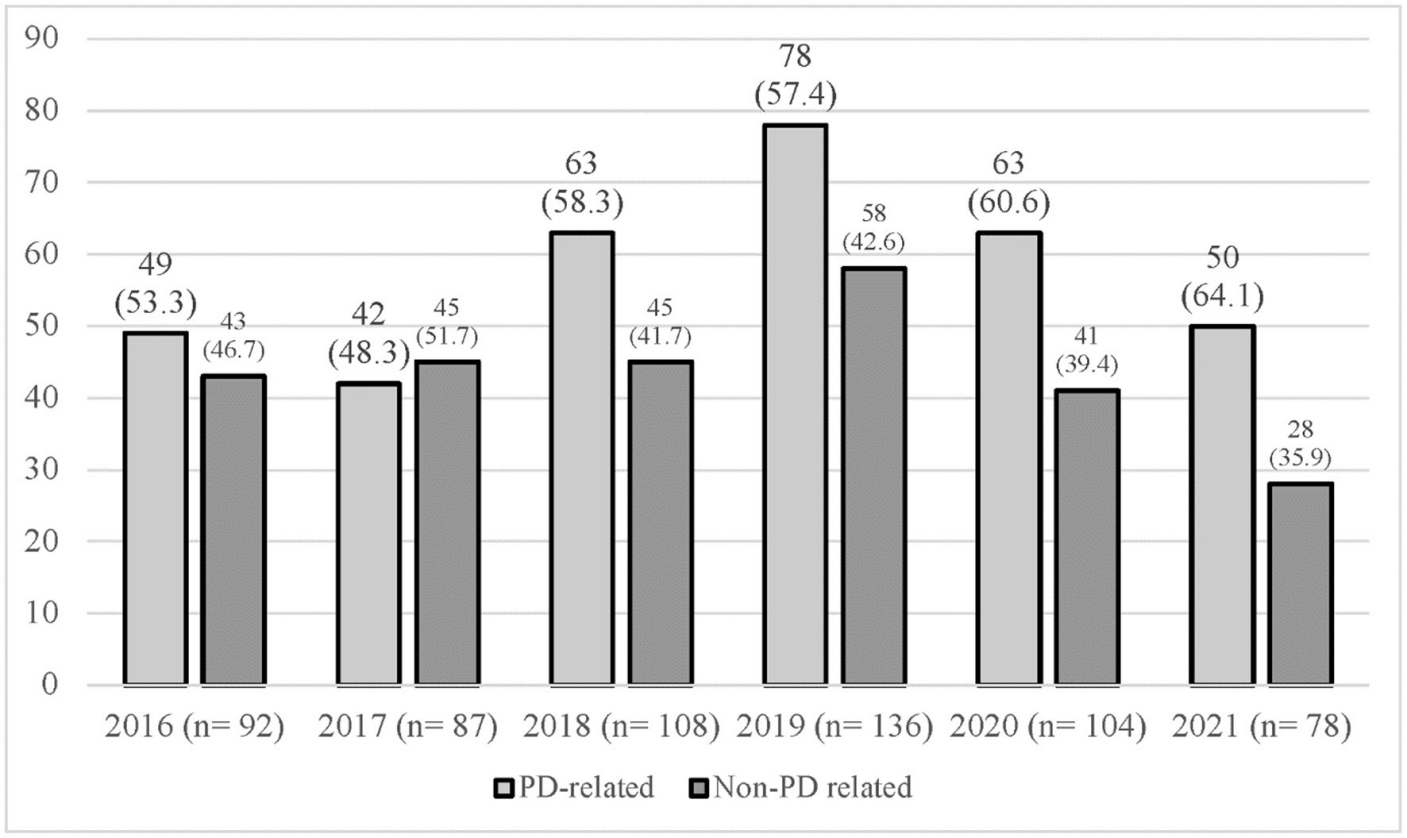
Causes of admission by year (2016–2021).

The trend of admission comparing between PD-related or non- PD-related causes of admission over the 6 years is shown in Figure 4. The number of PD admissions per year ranged from 78 (2021) to 136 (2019). In 2016 and 2017, there were similar frequencies of PD-related and non-PD-related admissions. In 2018 and 2019, there was a slightly higher frequency of PD-related admissions compared to non-PD-related admissions with a ratio of 1:1.3 and 1:1.4. However, there was a shift in the frequency of causes of admission in the years 2020 and 2021, with more admissions being PD-related compared to non-PD-related admissions at a ratio of 1:1.5 and 1:1.8.

### Comparison of factors and length of stay between PD- and non-PD-related causes

Factors significantly associated with different causes of admissions include race, the presence of comorbidities, duration of PD, and stage of PD. There were significantly more Chinese patients admitted for PD-related causes (*p* = 0.013) compared to other races. Almost all patients admitted for non-PD-related causes had comorbidities (97.3%) compared to PD-related causes (92.8%; *p* = 0.013). Patients admitted with PD-related causes had significantly longer PD duration compared to non-PD-related admissions [8.71 (SD 6.23) vs. 6.60 (SD 5.39); *p* < 0.001]. A significantly higher proportion of patients admitted for PD-related causes was in HY stage ≥3 (82.3%) compared to non-PD-related admissions (48.5%; *p* < 0.001). The length of stay was significantly longer for PD-related admissions [7.70 (SD5.89) vs. 6.42 (SD7.63); *p* = 0.02] (Table 3).

**Table 3:** Factors associated with admission causes.

	**PD-related causes *n* = 345**	**Non-PD-related causes *n* = 260**	**Statistical value**	***p*-value**
Age (years) mean (SD)	71.38 (10.85)	70.69 (8.89)	0.860^a^	0.390
Gender, *n* (%)		
Male	179 (51.9)	139 (53.5)	0.15^b^	0.700
Female	166 (48.1)	121 (46.5)		
Race, *n* (%)		
Malay	91 (26.4)	102 (39.2)	12.35^b^	0.002
Chinese	233 (67.5)	140 (53.8)		
Indian	21 (6.1)	18 (6.9)		
Comorbidities^*^, *n* (%)		
Yes	320 (92.8)	253 (97.3)	6.14^b^	0.013
No	25 (7.2)	7 (2.7)		
Staging of PD, *n* (%)			77.81^b^	< 0.001
< 3	61 (17.7)	134 (51.5)		
≥3	284 (82.3)	126 (48.5)		
PD duration (years) mean (SD)	8.71(6.23)	6.60 (5.39)	4.45^a^	< 0.001
Age of PD onset (years) Mean (SD)	62.61 (12.45)	63.92 (10.20)	−1.42^a^	0.156
Previous admission	268 (77.7)	214 (82.3)	1.96^b^	0.162
Length of stay (days) Mean (SD)	7.70 (5.89)	6.42 (7.63)	2.33^a^	0.020

^a^*t* test.

^b^Pearson chi-square.

SD, standard deviation.

^*^comorbidities (Medical, Orthopedic, Psychiatry, Others related, combination of ≥2 comorbid group).

### In-hospital death and complications related to admissions

Of the 605 admissions, 124 (20.5%) admissions had complications and 31 (5.1%) admissions resulted in in-hospital deaths. PD-related admissions accounted for 26 (83.9%) in-hospital deaths, while non-PD-related admissions accounted for the remaining five (16.1%) deaths. Infection was the top cause of death, which occurred in 29 out of 31 deaths, the most frequent being pneumonia (aspiration, hospital-acquired, orthostatic, and COVID-19). Death due to COVID-19 pneumonia occurred in three patients. Complications and causes of death are listed in Supplementary Table 2.

Deaths and complications occurred significantly more frequently in PD-related admissions compared to non-PD-related admissions (69.0 vs. 31.0%; *p* < 0.001). In-hospital deaths and complications were significantly associated with higher PD staging (*p* = 0.005) but not the duration of PD (*p* = 0.645). On logistic regression analysis, significant independent predictors for unfavorable hospital admission outcomes (death and complications) were emergency admissions (OR 2.49; *p* = 0.001) and respiratory infection (OR 2.40; *p* < 0.001) (Table 4).

**Table 4:** Predictors of unfavorable hospital admission outcome.

	**Admission outcome (death** + **complications)**		
**Factors**	**B**	**Adjusted OR (CI 95%)**	***P*** **-value**
Emergency admission (PD and Non-PD)	0.912	2.49 (1.429, 4.338)	0.001
Respiratory cause	0.879	2.40 (1.577, 3.677)	< 0.001
Age ≥50 years	0.993	2.44 (0.547, 10.904)	0.242
Comorbidities	−0.453	0.636 (0.288, 1.403)	0.262
Staging of PD ≥3	0.099	1.104 (0.687, 1.775)	0.682
Duration of PD >10 years	−0.371	0.690 (0.438, 1.087)	0.110

B, regression coefficient.

## Discussion

In this study, we provide a detailed description of the causes of hospitalization among patients with PD particularly on the most common causes, trends in causes of hospitalization, and complications arising from these admissions. In total, we identified 605 hospital admissions occurring in 259 patients with PD between 2016 and 2021, giving an average of 2.5 admissions per patient over the 6-year period. PD-related admissions were slightly more common than non-PD-related admissions. However, by analyzing the yearly trend of admission, the ratio of PD-related to non-PD-related admissions was found to be almost equal except between the years 2020 and 2021, where PD-related admissions exceeded non-PD-related admissions by almost 2-fold. This trend change occurred during the COVID-19 pandemic years, which indicates that admissions during the pandemic were likely to be due to worsening PD motor or non-motor symptoms, possibly due to missed clinical appointments due to movement control order restrictions during that period and/or patients' own preference to avoid hospitals during the pandemic or other factors such as a lack of physical activity or psychological distress (16). A study utilizing an online survey among patients with PD reported worsening PD symptoms during the pandemic as a result of psychological distress and reduced physical activity (17).

Emergency PD-related admissions accounted for 50.4% of all admissions, followed by emergency non-PD admissions which accounted for 23.8%. Elective admissions for PD-related causes contributed to only 6.7% of total admissions, while non-PD-related elective admissions contributed to 19.2% of all admissions. Elective PD-related admissions were mainly due to the levodopa challenge test, brain imaging, and deep brain stimulation surgery, while for elective admissions for non-PD-related causes, orthopedic procedures contributed to 38.8% of admissions. Although our center offers DBS, the number of patients who agree to undergo the procedure is still relatively small (only 3 admissions for DBS insertion), given the high cost, which could explain the small percentage of elective PD admissions recorded. Comparatively, an Australian study conducted over 5 years with more than 5,000 admissions had 400 admissions for DBS insertion [23]. The high frequency of admission for orthopedic procedures could be explained by the fact that 13.1% of patients in our study had orthopedic comorbidities. Besides that, patients with PD are also predisposed to bone and joint disorders such as frozen shoulder and postural disorders as a result of muscular rigidity and bradykinesia (18). It remains a challenge to determine to what extent PD contributes to the development of orthopedic problems due to the overlap in clinical symptoms.

For PD-related admissions, emergency admissions were more common than elective or planned admissions, while for non-PD-related admissions, emergency, and elective admissions were similar in frequency. Pneumonia was the most common cause and contributed to 45.6% of emergency PD admissions, followed by trauma (14.1%) and genitourinary infections (12.8%). Non-PD-related emergency admissions were most commonly due to infections or metabolic causes such as cellulitis, uncontrolled diabetes, and medication-induced electrolyte imbalance. Similar to our findings, a recent meta-analysis identified infections (urinary tract infection and pneumonia), gastrointestinal disorders, and falls/fractures as the top three causes of PD admissions (6). Previous studies on hospital admissions among patients with PD from other countries such as Ireland, India, Italy, Japan, Australia, and Singapore had similar findings, with the most common PD-related causes being infection (respiratory, urinary tract, sepsis, and aspiration pneumonia) and trauma due to falls with or without fracture (10, 19–24). Non-PD-related causes included myocardial infarction, arrhythmia, congestive cardiac failure, stroke, seizures, and renal failure (10). Combining all causes of admissions together irrespective of whether PD- or non-PD-related, respiratory infection remained the most common cause for admission in our study. The causes of respiratory infection were varied and included community-acquired pneumonia, aspiration pneumonia, hospital-acquired pneumonia, orthostatic pneumonia, and COVID-19 pneumonia.

Patients with PD are at a higher risk of being admitted due to a respiratory infection compared to the general population (25). They are predisposed to develop aspiration pneumonia as they are prone to dysphagia and silent aspiration due to multiple factors, which include delayed oral transit time as a result of lingual bradykinesia (26), pharyngo-esophageal dysmotility (27), and impaired cough reflex (25). A study that evaluated dysphagia in 119 patients with PD using flexible endoscopic evaluation of swallowing found that dysphagia was prevalent among patients with PD even when asymptomatic, whereby only 5% of patients with PD had normal deglutination. Aspiration was detected in 20% of patients with HY stage 2, and critical aspiration was observed in 16% of patients who were asymptomatic (28). A Japanese study that evaluated the relationship between dysphagia and depressive state in patients with PD found that the rates of dysphagia were 14.3% for HY stage 1, 50% for stage 4, and 100% for stage 5. Swallowing-related problems were an important cause of aspiration pneumonia and cause of death in that study (29). A recent study found that aspiration pneumonia increased the risk of mortality of PD over 2 years compared to controls (30). Our findings, therefore, highlight the importance of early screening for dysphagia, so that preventive measures for aspiration could be instituted, given the high frequency of hospital admissions and complications related to aspiration pneumonia.

There were significant differences in PD-related and non-PD-related admissions. Patients admitted for PD causes (direct and indirect) had a significantly higher disease stage (HY≥3) and a longer disease duration. In contrast, patients admitted with non-PD-related causes had more comorbidities. Patients admitted for PD causes also stayed longer than non-PD admissions. Taken together, our findings showed that PD-related admissions are more likely with a higher stage of PD and with longer disease duration and leading to a longer hospital stay. This finding is not unexpected, as patients are more likely to develop motor complications and worsening non-motor symptoms with disease progression. Thus, our findings highlight the need to optimize the medical care of patients with PD in order to prevent unnecessary hospital admissions related to suboptimal therapy and the morbidity associated with these admissions. The higher frequency of comorbidities in patients admitted for a non-PD cause is not surprising as the comorbidities were the most likely trigger factor for admission in these patients.

Repeated admissions contributed to 80% of all admissions in our study. As we did not perform a detailed analysis of each repeated admission, we were unable to determine if these admissions were related to the first admission or other causes. However, given the mean age of our patients which was 72.4 years, and as 95% of them had at least one comorbidity, it is not unexpected to have such a high frequency of repeated admissions. Studies have shown that those with a history of hospitalization are more likely to be re-hospitalized (31). Another study revealed that disease severity, presence of comorbidities, DBS, motor fluctuations, and poor quality of life predisposed to higher rates of repeated hospitalizations (32). Hospitalizations led to deterioration in Parkinson's symptoms with poor motor outcomes in 20% of patients and 44% never returned to their baseline functional status prior to hospital admission (31, 33). This deterioration in functional status was irrespective of the cause for admission (32). With the rise in the aging population in Malaysia, admission costs are likely to rise, and with repeated admissions, patients and the healthcare system would incur a significant economic burden (33).

Complications occurred in 20.5% of all admissions, while death occurred in 5.1% of all admissions over the 6 years. The most common cause of death was pneumonia, three of which were due to COVID-19 pneumonia. Expectedly, there were significantly higher rates of death and complications among PD-related admissions compared to non-PD-related admissions and in patients with higher HY stages (≥3). A study conducted in Portugal found that 150 out of 1,525 PD admissions between 2008 and 2014 resulted in death in 9.8%, which was slightly higher than our findings (34). Significant independent predictors of unfavorable hospital admission outcomes (death and complications) in our study were admissions due to emergency causes and respiratory infection. Similar to our findings, a study conducted in the UK found that patients with PD are at increased risk of mortality with the commonest cause of death being pneumonia (35).

There were 11 COVID-19 admissions in the years 2020 and 2021 but four were excluded due to missing data. Of the seven COVID-19 pneumonia included in this study, all had comorbidities and advanced age (mean age 72.7 years). All seven admissions had complications and three resulted in death, reinforcing the high morbidity and mortality associated with COVID-19 in patients with PD. It is interesting to note that all deaths except one, taking into account data not included for analysis, occurred in the year 2021, when the delta variant of SARS-CoV-2 was the most prevalent cause of COVID-19 infections in Malaysia and globally (36).

Collectively, the findings of our study add to the existing literature on hospital admissions among patients with PD, which could be generalized to other populations. While retrieving data for this study, we were able to determine the approximate number of hospitalized (relative to total adult admissions) patients with PD, giving a hospitalization rate of 0.3% or 3 in 1,000. An epidemiological study conducted in Spain reported a hospitalization rate of 64.2 per 100,000 cases (0.6%) among patients with PD (37), which was slightly higher than ours. Respiratory infection remained the leading cause of death and admission and contributed to the majority of emergency admissions in patients with PD. This highlights the importance of incorporating respiratory training interventions in the management of patients with PD. More attention should be given to optimizing therapy for motor and non-motor symptoms during clinical visits, especially in patients with advanced disease. Efforts should also be taken to prevent COVID-19 infections by adhering to guidelines in order to prevent transmission through vaccination.

## Study limitations

We acknowledge that there are several limitations to our study. First, data collection was conducted during the COVID-19 pandemic which had caused restrictions in accessing the medical record office during the movement control order. Second, there were missing files and missing data and some patients did not have a clear diagnosis of idiopathic PD as they were diagnosed at a different center without any details of the disease. Third, we had to determine the stage of PD based on the functional status documented or the most recent clinical visit, as the staging of PD was not objectively documented in selected case notes. Ideally, detailed objective assessments should be done using evaluation tools such as validated scales and questionnaires. Fourth, the ICD coding system required refinement as many cases identified with the G20 coding system for PD included secondary PD, drug-induced parkinsonism, and essential tremors resulting in many shortlisted case notes being rejected (38).

## Strength and recommendations

Still, we believe that our study has several strengths. First, to the best of our knowledge, this is the first study in Malaysia to provide a detailed analysis of the causes and factors associated with admissions and complications among patients with PD. Second, in order to avoid variations in the classification of causes of admission, and to ensure homogeneity with other studies, we based our classification on two studies, which similarly divided the causes of admission into PD-related and non-PD-related, and further divided PD-related causes into direct and indirect causes (10, 15). In addition, as our study period included the COVID-19 pandemic years, we were able to compare the trend of admissions prior to and during the pandemic. In the future, detailed prospective studies should be conducted to identify important risk factors associated with hospital admissions among patients with PD.

## Conclusion

In conclusion, our study showed that emergency PD admissions were the most common cause of admissions among patients with PD. Pneumonia was the most frequent cause of admissions and a leading cause of death related to PD admissions. Our findings highlight the importance of optimizing the treatment of parkinsonism adequately, with detailed attention toward the management of comorbidities and recognizing aspiration risk, and incorporating interventions such as respiratory training and swallowing assessment so that hospital admissions can be prevented. This in turn could hopefully avoid a myriad of complications, such as deterioration in clinical staging related to hospital admissions, which would further impact their quality of life and increase the risk of re-hospitalization.

## Data availability statement

The raw data supporting the conclusions of this article will be made available by the authors, without undue reservation.

## Ethics statement

The studies involving human participants were reviewed and approved by the Universiti Kebangsaan Malaysia (UKM) Medical Research Ethics Committee (research code: JEP-2021-263). Written informed consent from the patients/participants or patients/participants' legal guardian/next of kin was not required to participate in this study in accordance with the national legislation and the institutional requirements.

## Author contributions

All authors listed have made a substantial, direct, and intellectual contribution to the work and approved it for publication.
