# Supplementary material for: Causes, factors, and complications associated with hospital admissions among patients with Parkinson's disease
Source: Front Neurol. 2023 Mar 7;14:1136858. doi: 10.3389/fneur.2023.1136858 (PMC10027758; doi:10.3389/fneur.2023.1136858)
Supplement: Supplementary file 1 [file Table_1.pdf]

Supplementary Table 1: Causes of Repeated Admissions

**PD-related causes n= 268 (55.6%)**				**Non-PD-related causes n= 214 (44.4%)**			
Emergency n= 238 (88.8)		Elective n =30 (11.2)		Emergency n= 114 (53.3)		Elective n=100 (46.7)	
**Direct PD-related n (%)**				Cardiac	28 (24.6)	Ortho procedures[b]	41 (41.0)
Motor symptoms	18 (7.6)	Procedures[a]	24 (80.0)	End stage Renal disease	2 (1.8)	Non-ortho procedures[c]	59 (59.0)
Non motor symptoms	8 (3.4)	Medication adjustment	6 (20.0)	Stroke	6 (5.3)		
Neuro-psychiatric symptoms	2 (0.8)			Anemia (bleed, chronic illness)	26 (22.8)		
Medication adverse effects	13 (5.5)			Infection/ Metabolic (catheter, gangrene, abscess)	52 (45.6)		
**Indirect PD-related**							
Sepsis (total)	158 (66.4)						
Respiratory	108 (45.4)						
Genito-urinary	31 (13.0)						
Gastro-intestinal	3 (1.3)						
Pressure sores	16 (6.7)						
Trauma (fall, fracture)	31 (13.0)						
Delirium	1 (0.4)						
Electrolyte imbalance	3 (1.3)						
Others[d]	4 (1.7)						

[a] procedures: levodopa challenge, brain imaging
[b]orthopaedic procedures such as nerve block, imaging, surgery
[c] non-orthopaedic procedures such as catheter insertion for dialysis, fistula creation, cataract surgery and angiogram
[d]Others; PEG tube infection

Supplementary Table 2: Complications and causes of death (2016-2021)

**Complications;** *n =124 (20.5%)*	
Hospital acquired infection	47 (37.9)
Clinical stage deterioration	35 (28.2)
Delirium	32 (25.8)
Others (anaemia, ACS, AKI, hematoma)	31 (25.0)
Pressure sore	22 (17.7)
Acute urinary retention	17 (13.7)
Shock	7 (5.6)
**Death,** *n=31 (5.1%)*	
Infection related (Sepsis)	29 (93.5)
(HAP/aspiration pneumonia/orthostatic pneumonia)	17 (58.6)
(Multiple infected pressure sore/AKA stump/septic arthritis/gangrene	5 (17.2)
Severe urosepsis	3 (10.3)
COVID-19 pneumonia	3 (10.3)
Severe sepsis due to bowel ischemia	1 (3.4)
Sudden cardiac death	2 (6.5)

ACS, acute coronary syndrome; AKI, acute kidney injury; HAP, hospital acquired pneumonia; AKA, above knee amputation
